# Biodeterioration Risk Threatens the 3100 Year Old Staircase of Hallstatt (Austria): Possible Involvement of Halophilic Microorganisms

**DOI:** 10.1371/journal.pone.0148279

**Published:** 2016-02-17

**Authors:** Guadalupe Piñar, Dennis Dalnodar, Christian Voitl, Hans Reschreiter, Katja Sterflinger

**Affiliations:** 1 Department of Biotechnology, VIBT-Vienna Institute of BioTechnology, University of Natural Resources and Life Sciences, Vienna, Austria; 2 Prehistoric Department, Museum of Natural History, Vienna, Austria; Medical University Graz, AUSTRIA

## Abstract

**Background:**

The prosperity of Hallstatt (Salzkammergut region, Austria) is based on the richness of salt in the surrounding mountains and salt mining, which is documented as far back as 1500 years B.C. Substantial archaeological evidence of Bronze and Iron Age salt mining has been discovered, with a wooden staircase (1108 B.C.) being one of the most impressive and well preserved finds. However, after its discovery, fungal mycelia have been observed on the surface of the staircase, most probably due to airborne contamination after its find.

**Objective:**

As a basis for the further preservation of this valuable object, the active micro-flora was examined to investigate the presence of potentially biodegradative microorganisms.

**Results:**

Most of the strains isolated from the staircase showed to be halotolerant and halophilic microorganisms, due to the saline environment of the mine. Results derived from culture-dependent assays revealed a high fungal diversity, including both halotolerant and halophilic fungi, the most dominant strains being members of the genus *Phialosimplex* (synonym: *Aspergillus*). Additionally, some typical cellulose degraders, namely *Stachybotrys* sp. and *Cladosporium* sp. were detected. Numerous bacterial strains were isolated and identified as members of 12 different genera, most of them being moderately halophilic species. The most dominant isolates affiliated with species of the genera *Halovibrio* and *Marinococcus*. Halophilic archaea were also isolated and identified as species of the genera *Halococcus* and *Halorubrum*. Molecular analyses complemented the cultivation assays, enabling the identification of some uncultivable archaea of the genera *Halolamina*, *Haloplanus* and *Halobacterium*. Results derived from fungi and bacteria supported those obtained by cultivation methods, exhibiting the same dominant members in the communities.

**Conclusion:**

The results clearly showed the presence of some cellulose degraders that may become active if the requirements for growth and the environmental conditions turn suitable; therefore, these microorganisms must be regarded as a threat to the wood.

## Introduction

Hallstatt is located in the Austrian region “Salzkammergut” (Upper Austria), and is one of the most important archaeological sites of the Old World. For this reason, the region was designated as a UNESCO World Cultural Heritage site in 1997. The prosperity of the region dates back to the Bronze Age and was based on the richness of salt in the surrounding mountains. The salt mining in the galleries is documented as far back as 1500 years B.C.

As a result of the extremely high salt concentrations inside the mine galleries, the remains of the Bronze and Iron Age salt miners have been well preserved, and prehistoric findings have been reported since 1607 [1]. Research to uncover the history of Hallstatt began in earnest in the first half of the 19^th^ Century, but it was not until 1960 that an exhaustive excavation of the galleries was initiated, resulting in the discovery of several important archaeological findings of wood, pelt, skin, bast fibres, clothes, textiles, tools, in addition to food and excrement remains [2].

**Fig 1:**
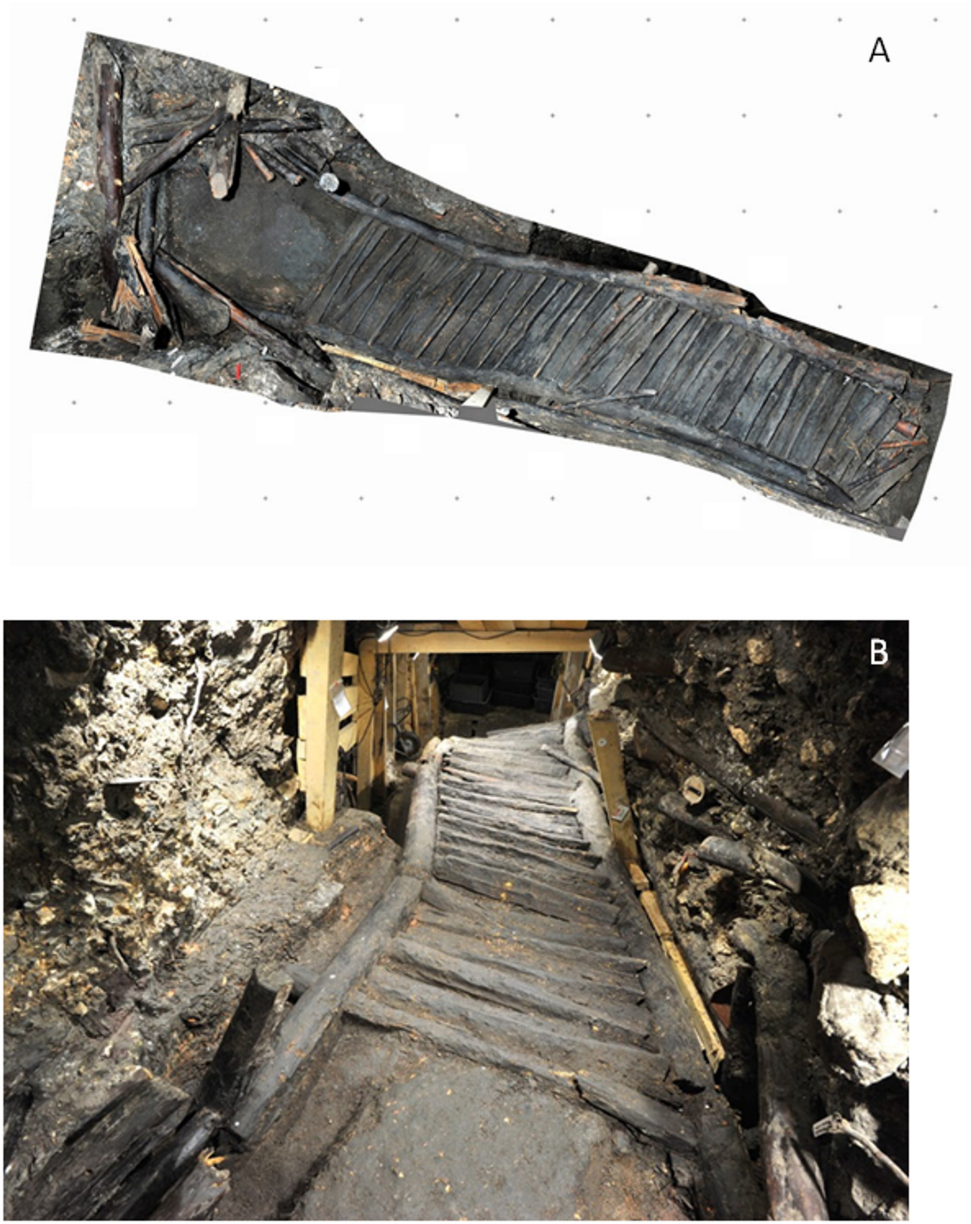
The staircase of Hallstatt. The 3100 years old staircase is 8 m long and 1.20 wide, and is representative of the construction typical of the Bronze Age (C: NHM/A. Rausch) (**A**). After its discovery, a wooden shelter was built over the staircase to ensure its preservation (C: NHM/A. Rausch) (**B**).

One of the most important discoveries was a wooden staircase, built in the year 1108 B.C. to span two floor levels in a mine gallery. The staircase had been in use for approximately 100 years, until it was buried by a landslide in 1000 B.C. [1]. The staircase is 8 m long and 1.20m wide, and is a fine example of Bronze Age construction (Fig 1a). After its discovery, the chamber with the staircase was timbered to ensure its preservation (Fig 1b). However, the continuous earth movements occurring in this area necessitated the removal of the staircase from the mine, in order to protect it. The stairs were removed one by one and brought to the Museum of Natural History (NHM) in Vienna, Austria, the last arriving in spring 2014. Since then, numerous investigations have been performed by researchers of the museum and the university, including scanning, computed tomography, chemical and biological analyses as well as documentation (Personal communication, NHM). Finally, in February 2015, the staircase was installed in a huge showcase within the mine and is now part of the guided tours through the show-mine in the “Salzwelten”, Hallstatt.

The structure and stability of the wooden staircase were fully intact at the time of its discovery [3], due to the suppression of common wood-decaying fungi by the salt. However, since 2000, it is well known that fungi are active inhabitants of solar salterns [4] and new species, like *Debaryomyces hansenii*, *Hortaea werneckii*, *Wallemia ichthyophaga* and *Phialosimplex salinarum* have been isolated from natural hypersaline environments around the globe [5, 6].

**Fig 2:**
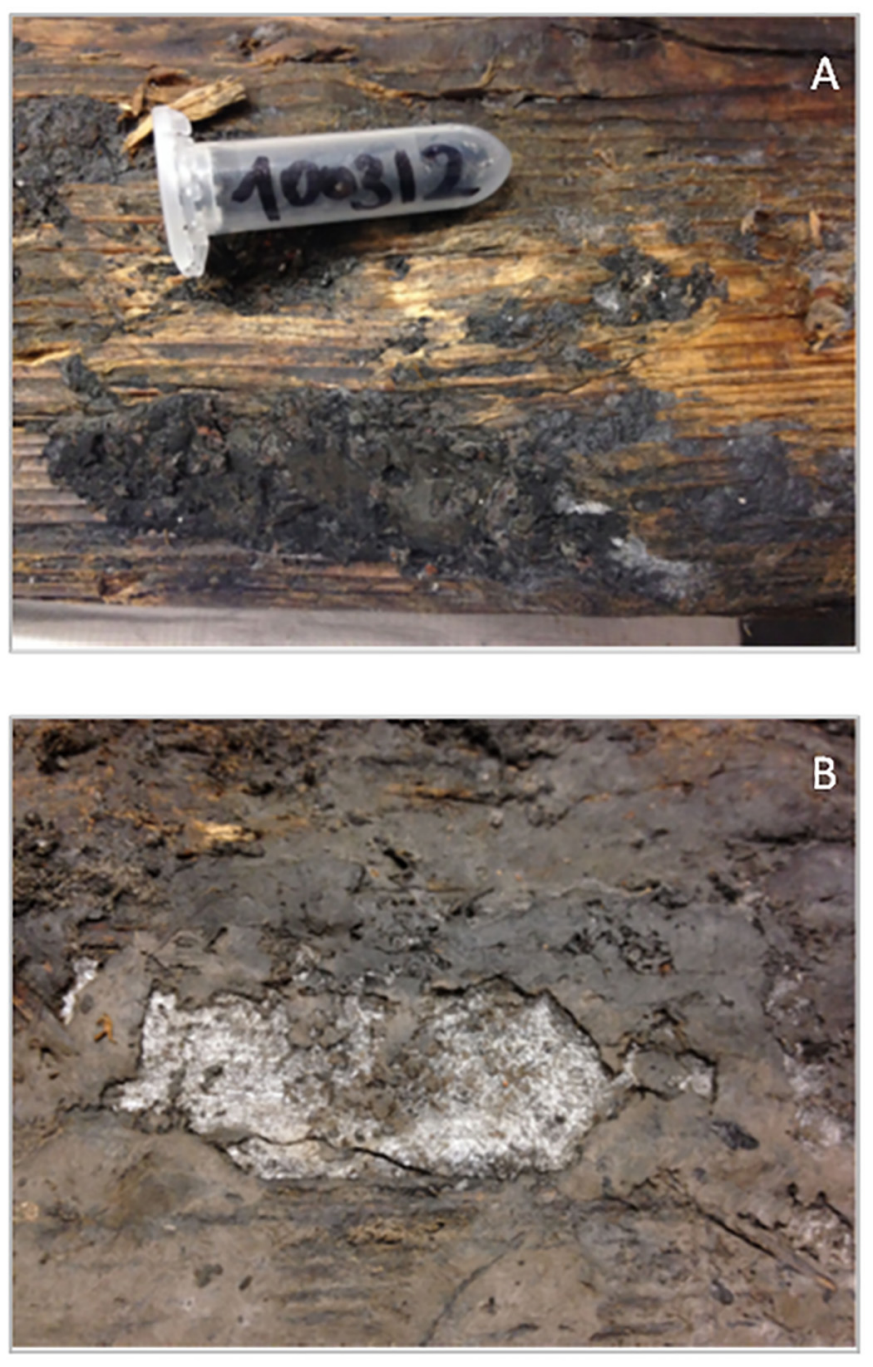
Stairs surface. Fungal mycelia developing on the wood and on the old dark deposits on the surface of the stairs (**A**). Evolution of salt crusts due to the storing conditions promoting local variations in the salt content on the stairs (**B**).

Subsequent to the discovery and excavation of the staircase, and upon its relocation to the NHM in Vienna, fungal mycelia started to develop on the wood and on the old dark deposits on the surface of the stairs (Fig 2a). Researchers thought that this might be due to the storage conditions, even though, for the duration of the scientific analyses, the stairs were single wrapped into non-woven textile material and stored on shelves in the NHM. Nevertheless, these storage conditions allowed the salts to wick in the wood, enabling the evolution of salt crusts on the surface (Fig 2b) and promoting local variations in the salt distribution on the stairs. At this point, the contamination of the wood with common microflora, not adapted to high salinity, and having the capability to degrade lignin and cellulose could become a serious problem. It has already been found that filamentous fungi are able to cause deterioration in books, paper documents and wooden statues [7–10].

Thus, as a basis for the further preservation of this wooden staircase, it was necessary to analyze the micro-flora thriving on the wood and on the old patina in order to check for possible biodegradative microorganisms that may become active if the requirements for growth and the environmental conditions turn suitable. To this end, a polyphasic approach was applied, comprised of enrichment cultures conducted on media containing different NaCl concentrations, ranging from 3–20% (w/v), and the screening of the isolated strains by two different PCR-genotyping based methods: the random amplification of polymorphic DNA (RAPD) analysis for Bacteria and Archaea and the amplification of the cellobiohydrolase gene (*cbh*-I) for Fungi. In addition, molecular analyses including DGGE-fingerprinting and sequencing were conducted in order to complement the data obtained by culture-dependent methods. Finally, this study summarized recommendations concerning the conservation of the staircase.

## Materials and Methods

### Sampling and object

Samples were taken from a wooden staircase which had been discovered in 2003 on the site named “Christian von Tusch Werk” in a salt mine in the Austrian region of “Salzkammergut” (Upper Austria) (S1 Fig). The whole sampling procedure was performed at the NHM, Vienna. Mag. H. Reschreiter and the Museum of Natural History (NHM) issued the permission for sampling. HR supervised the whole sampling procedure.

Samples were collected from three representative stairs by non-invasive sampling using commercial contact plates as follows: two plates of 2% (w/v) Malt Extract Agar (MEA) and two Dichlorane Glycerol + chloramphenicol (DG18) agar (55 mm, Heipha, Eppelheim, Germany) per stair. The plates were opened and the agar surface was evenly pressed onto the dry surface of the wood in areas were mycelia was visible for a maximum of 5 seconds. Afterwards the plates were closed and brought to the laboratory for incubation.

In addition, several samples were taken by minimal-invasive sampling using sterile scalpels. These samples were scraped off and collected from some areas showing visible mycelia and from the old covering dark biofilm. The number of the sample (425, 1003 and 13216) indicates the labelling number of the stair from which they were obtained. In addition, from sample 13216, a small piece of a salt crust was taken. Scrapes taken from the stairs Nr. 1003 and 13216, respectively, were pooled independently to obtain a single sample per stair for further analyses. Then, each pool was divided into 5 aliquots for: 1) molecular analyses, 2) the direct isolation of fungi, 3) enrichment cultures for the isolation of halotolerant/halophilic bacteria and archaea in 3% (w/v) NaCl medium, 4) 10% (w/v) NaCl medium and 5) 20% (w/v) NaCl medium. For sample Nr. 425, less material could be obtained, and so the corresponding scrapes were pooled and divided only into two aliquots: the first used for molecular analyses and the second for the direct isolation of fungi.

### Cultivation assays

For the isolation of fungi, two different approaches were applied. The first consisted of the incubation of the utilized commercial contact plates mentioned above, containing MEA and DG18, the first medium used for the isolation and detection of yeast and fungi, and the second being a selective low water activity (aw) medium for xerophilic molds.

In addition, three different agar media were prepared in the laboratory and used for the direct isolation of fungi from the wood scrapes: Dichloran Rose-Bengal Chloramphenicol Agar (DRBC), Malt Extract Agar (MEA) and Salt Agar [(SA), containing 0.1% (w/v) malt extract, 0.67% (w/v) Nitrogen base, 5% (w/v) glucose, 2% (w/v) agar, added to 10% (w/v) NaCl]. To avoid bacterial growth, the last two media were supplemented with 100 μg ml^-1^ ampicillin and 25 μg ml^-1^ streptomycin (Sigma). The scrapes aliquots of each stair destined for direct fungal isolation were suspended in 2.5 ml of sterile 0.9% (w/v) NaCl solution by vortexing. From each solution, 3 x 100 μl were directly pipetted on DRBC, MEA and SA media, resulting in a total of 27 petri dishes. These plates and the 12 obtained contact plates were incubated aerobically at room temperature (20–22°C) for between 9 and 30 days, depending on the growth of fungi. Grown colonies were transferred to fresh plates containing MEA and SA media to obtain further pure isolates. The pure isolates were then incubated at room temperature (20–22°C) until the mycelium was sufficiently grown for DNA-extraction (20–48 days).

For the isolation of Bacteria and Archaea, enrichment cultures were started using three different media: Trypticase Soy Agar (TSA) supplemented with NaCl (3% w/v) and MgSO4·7H_2_O (2% w/v) [11], Maintenance Medium (HMM, 10% w/v NaCl) [12] and M2 medium (20% w/v NaCl) [13]. Enrichments were conducted with scrapes aliquots of 0.1–0.130 g and 0.4–0.470 g for samples 13216 and 1003, respectively. The aliquots were deposited in 300 ml Erlenmeyer flasks containing 30 ml medium. To avoid fungal growth, media were supplemented with 50 μg ml^-1^ cycloheximide (Sigma). Flasks were incubated aerobically at 28°C by shaking at 180rpm (Thermo Scientific Equipment MaxQ 8000, Germany). Over a total period of 6 weeks, weekly aliquots of 100 μl enrichments were serial-diluted and plated onto the same solid media (without cycloheximide). Plates were incubated aerobically at room temperature (20–22°C) for 2–10 weeks, depending on the growth of the microorganisms. The colony morphology was examined on an Olympus SZX9 phase contrast microscope. Colonies showing different morphology and appearance were transferred to new culture plates to obtain pure cultures. Pure isolates were cultivated in fresh media until exponential growth occurred, to be stored finally in 70% (v/v) glycerol at -80°C for preservation.

### DNA extraction and genotyping of the isolated microflora

DNA extraction of pure fungal strains was performed according to the procedure described by Sert and Sterflinger [14], which relies on chemical as well as mechanical disruption of cells, by using glass beads and the Fast Prep FP120 Ribolyzer (MP Biomedicals, Illkrich, France), and the recovery of DNA by ethanol precipitation. Genomic bacterial DNAs were extracted according to the protocol provided by Ausubel *et al*. [15], which relies on a chemical disruption of cells, the removal of cell wall debris, polysaccharides and proteins by precipitation with CTAB, and the recovery of the DNA by ethanol precipitation.

All PCR reactions were performed with the 2x PCR Master Mix from Promega (Vienna, Austria) [50 units ml^-1^ of TaqDNA Polymerase supplied in an appropriate reaction buffer (pH 8.5), 400 μM dNTP’s, 3 mM MgCl2], which was diluted to 1x, and 12.5 pmol μl^-1^ of each primer (stock: 50 pmol μl^-1^, VBC-Biotech, Austria) were added. In a total volume of 25 μl, 400 μg ml^-1^ BSA (stock: 20 mg ml^-1^; Roche, Diagnostics GmbH, Germany) and 2.5 μl DNA template were added (average concentration of DNA was 50–60 ng per reaction). All PCR reactions were executed in a BioRad C1000 Thermal Cycler.

The genotyping of fungal strains was performed by using the polymorphism of the cellobiohydrolase gene (*cbh*-I), as described by Kraková *et al*. [10]. The primer-pair: CbH-fw–TCG AYG CSA ACT GGC GCT GG and CbH-rv–TTG GCY TCC CAG AYA TCC ATC TC was used. The cycling program consisted of 5 min at 95°C, followed by 35 cycles of [95°C, 1 min; 55°C, 1 min and 72°C, 30 sec] and a final elongation step for 8 min at 72°C. The genotyping of bacterial and archaeal strains was conducted with random amplified polymorphic DNA-polymerase chain reaction (RAPD-PCR) analyses, using the single primer D11344, being 23 nt in length, as described by Ripka *et al*. [16]. The cycling program was 4 cycles of [94°C, 5 min; 40°C, 5 min and 72°C, 5 min; low stringency amplification], 30 cycles of [94°C, 1 min; 55°C, 1 min and 72°C, 2 min; high stringency amplification] and a final elongation step for 10 min at 72°C [17].

The entire reaction batches were run with 4 μl loading dye solution (Fermentas) in a 2% (w/v) agarose gel for ~130–160 min at 70 V, stained in an ethidium bromide solution (1 mg ml^-1^; stock 10 mg ml^-1^) for 30 min and visualized by a UVP documentation system (BioRad Transilluminator, Universal Hood; Mitsubishi P93D-printer). The GeneRulerTM 100 bp DNA ladder (Fermentas) was used as a size marker.

### DNA sequencing and phylogenetic analyses of isolated strains

For sequencing of fungal isolates, the primer pair ITS1 [18] and NL4 [19] were used. The thermocycling program used was as follows: 1 min at 98°C, followed by 35 cycles consisting of [98°C, 30 sec; 60°C, 30 sec and 72°C, 105 sec] followed by a final extension step for 2 min at 72°C.

For sequencing of bacterial isolates, 16S rRNA fragments were amplified using the forward primer 27f and the reverse primer 1492r [20]. The thermocycling program used was as follows: 5 min at 95°C, followed by 30 cycles consisting of [95°C, 1 min; 55°C, 1 min and 72°C, 2 min] followed by a final extension step for 5 min at 72°C.

For sequencing of archaeal isolates, 16S rRNA fragments were amplified using the forward primer ARC344 and the reverse primer ARC915 [21]. The following thermocycling program was used: 5 min at 95°C, followed by 40 cycles consisting of [95°C, 1 min; 60°C, 1 min and 72°C, 1 min] with a final extension step for 5 min at 72°C.

All PCR products obtained were purified using the QIAquick PCR Purification Kit (Qiagen) and analyzed by electrophoresis in 2% (w/v) agarose gels. PCR products were externally sequenced by Sanger sequencing (GATC Biotech, Germany). Comparative sequence analyses were performed by comparing pairwise insert sequences with those available in the online databases provided by the National Center for Biotechnology Information using the BLAST search program [22]. The sequences retrieved from all isolated strains have been deposited in the NCBI nucleotide database under the accession numbers listed in Tables 1 and 2.

**Table 1:** Phylogenetic affinities of the ITS coding sequences amplified from pure fungal strains isolated from the staircase of Hallstatt.

Strain	Isolation method*	Cultivation agar	Length	Closest identified phylogenetic relatives [EMBL accession numbers]	Similarity(%)	AccessionNo
**Sample 425**						
HF1	CP-GD18	SA	[561]	Uncultured fungus clone SXF24 [JQ410077] detected at an archaeological excavation site in Yicheng, Shanxi, China. /*Phialosimplex salinarum* [KF274692, KF274685] isolated from saline water from a salt mine.	99/96	KR081397
HF2	CP-GD18	MEA	[518]	*Aspergillus* sp. [AY373890, FR848828, FR839678].	99	KR081398
HF22	CP-MEA	MEA	[629]	*Exophiala* sp. strain 4-11c [KC790479] from cadmium contaminated soil.	99	KR081415
HF29	DP-SA	SA	[563]	*Phialosimplex salinarum* [KF274692, KF274685] isolated from saline water from a salt mine.	99	KR081418
HF39	DP-SA	SA	[572]	*Phialosimplex salinarum* [KF274692, KF274685] isolated from saline water from a salt mine.	99	KR081422
**Sample 1003**						
HF3	CP-MEA	MEA	[539]	*Nectria pseudotrichia* isolate 0911ARD7G1 [LN808859] from air sample in a cave, Spain.	94	KR081399
HF4	CP-MEA	MEA	[532]	*Stachybotrys* sp. [KF367489, KC305345, KC787692].	99	KR081400
HF5	CP-MEA	MEA	[523]	Uncultured *Cladosporium* clone 16w3 [JX860445] community structure of wood decay fungi. /*Cladosporium* sp. [FJ770061, FJ755820, DQ092532].	99/98	KR081401
HF6	CP-GD18	MEA	[548]	*Aspergillus ochraceus* isolate T3-16 [GU134890] from plant habitats.	99	KR081402
HF7	CP-GD18	SA	[561]	*Wallemia muriae* strain EXF 8592 [KJ409919] from house dust and indoor air.	99	KR081403
HF8	CP-GD18	MEA	[965]	*Brachycladium papaveris* strain Min-l_HU [GQ995478] isolated from leafs.	98	KR081404
HF9	CP-GD18	MEA	[775]	*Penicillium chrysogenum* strain B-090 [KJ939428] cellulase and hemicellulase producing fungi isolated from compost and litter.	99	KR081405
HF10	CP-GD18	MEA	[618]	*Sterigmatomyces halophilus* CBS 4609 [NR_073302].	99	KR081406
HF11	CP-GD18	MEA	[569]	*Aspergillus restrictus* isolate NRRL 148 [EF652040].	99	KR081407
HF12	CP-GD18	MEA	[775]	*Penicillium griseofulvum* strain NRRL 3523 [DQ339549].	99	KR081408
HF16	CP-MEA	MEA	[688]	Uncultured fungus clone CMH410 [KF800501] from indoor environments. /*Verrucocladosporium dirinae* strain CBS 112794 [EU040244].	99/98	KR081411
HF19	CP-MEA	MEA	[592]	*Phlebia livida* subsp. *tuberculata* isolate FCUG3157 [HQ153427].	98	KR081412
HF20	CP-GD18	MEA	[539]	*Nectria pseudotrichia* isolate 0911ARD7G1 [LN808859] from air sample in a cave, Spain.	94	KR081413
HF21	CP-MEA	MEA	[542]	*Nectria pseudotrichia* isolate 0911ARD7G1 [LN808859] from air sample in a cave, Spain.	94	KR081414
HF31	DP-SA	SA	[563]	*Phialosimplex salinarum* [KF274692, KF274685] isolated from saline water from a salt mine.	99	KR081419
HF33	DP-SA	SA	[563]	Uncultured fungus clone SXF24 [JQ410077] detected at archaeological excavation site in Yicheng, Shanxi, China. /*Phialosimplex salinarum* [KF274692, KF274685] isolated from saline water from a salt mine.	99/97	KR081420
**Sample 13216**						
HF13	CP-MEA	MEA	[746]	*Engyodontium album* isolate AHF [KC311469] isolated from a wall in Austria.	99	KR081409
HF14	CP-GD18	MEA	[524]	*Cladosporium sphaerospermum* strain TFSe2 [HQ443241].	99	KR081410
HF24	DP-DCRB	MEA	[508]	*Pseudotaeniolina globosa* isolate ICP1002 [KC311489] isolated from soils in Austria.	99	KR081416
HF25	DP-DCRB	MEA	[531]	*Aspergillus versicolor* strain isolate DFFSCS010 [JX156356] deep-sea fungi from the South china sea.	99	KR081417
HF37	DP-SA	SA	[560]	Uncultured fungus clone SXF24 [JQ410077] detected at archaeological excavation site in Yicheng, Shanxi, China. /*Phialosimplex salinarum* [KF274692, KF274685] isolated from saline water from a salt mine.	99/96	KR081421

* CP: contact plates; DP: direct plating of wood scrapes suspended in 2.5 ml of sterile 0.9% (w/v) NaCl solution.

**Table 2:** Clustering of bacterial and archaeal strains according to RAPD-PCR and phylogenetic classification of the isolated bacteria and archaea.

[NaCl](%)	Strains grouped by RAPD *	Closest phylogenetic relatives [EMBL accession numbers]	Phyla	Similarity (%)	Accession No.
**Sample 1003**					
**3**	*HB4*	*Bacillus licheniformis* strain 55N2-7 [JN366716].	*Firmicutes*	99.0	KR054055
**3**	*HB12*, HB5, HB23	*Staphylococcus equorum* strain PT32c [HQ439552].	*Firmicutes*	99.0	KR054056
**3**	*HB13*, HB6	Uncultured bacterium clone R3-108 [EF363039] from deep saline fluid from the German continental deep drilling (KTB) pilot hole. */Idiomarina* sp. TBZ4 [HQ190037] Urmia Lake in Iran.	*Proteobacteria* (Gamma-class)	99.0/99.0	KR054057
**3**	*HB14*	*Staphylococcus hominis* subsp. *novobiosepticus* strain ALK519 [KC456583] from activate sludge.	*Firmicutes*	99.0	KR054058
**3**	*HB45*	*Janibacter cremeus* strain HR08-44 [NR_114380] an actinobacterium isolated from sea sediment.	*Actinobacteria*	99.0	KR054059
**10**	*HB9*, *HB10*, HB19	Uncultured *Halovibrio* sp. clone BPS_CK84 [HQ857607] from petroleum hydrocarbon contaminated gradient in a saline alkaline soil. /*Halovibrio* sp. K12-34B [KC309459] Isolated from temporary saline tidal pools.	*Proteobacteria* (Gamma-class)	98.0/98.0	KR054060KR054061
**10**	*HB11*, HB43	*Marinococcus halophilus* strain AX17 [KJ873908] from soil samples of Qaidam Basin, China.	*Firmicutes*	99.0	KR054062
**10**	*HB20*, HB7, HB37	*Marinococcus halophilus* strain AX17 [KJ873908] from soil samples of Qaidam Basin, China.	*Firmicutes*	99.0	KR054063
**10**	*HB21*	Uncultured bacterium clone SN180 [JQ825066] from Hexi corridor saline-alkali soil. /*Marinococcus halophilus* strain AX17 [KJ873908] from soil samples of Qaidam Basin, China.	*Firmicutes*	99.0/99.0	KR054064
**10**	HB40, *HB42*	*Janibacter cremeus* strain HR08-44 [NR_114380] an actinobacterium isolated from sea sediment.	*Actinobacteria*	98.00	KR054065
**10**	*HB44*	*Planococcus* sp. NPO-JL-69 [AY745836] pigmented heterotrophic bacteria in marine environments.	*Firmicutes*	99.00	KR054066
**20**	*HB16*	*Marinococcus halophilus* strain AX18 [KJ873909] from soil samples of Qaidam Basin, China.	*Firmicutes*	99.0	KR054067
**20**	*HB29*	Uncultured *Halovibrio* sp. clone BPS_CK84 [HQ857607] petroleum hydrocarbon contaminated gradient in a saline alkaline soil. /*Halovibrio* sp. K12-34B [KC309459] Isolated from temporary saline tidal pools.	*Proteobacteria* (Gamma-class)	98.0/98.0	KR054068
**20**	*HB30*, HB32, HB66	Uncultured *Halovibrio* sp. clone BPS_CK84 [HQ857607] petroleum hydrocarbon contaminated gradient in a saline alkaline soil. /*Halovibrio* sp. K12-34B [KC309459] Isolated from temporary saline tidal pools.	*Proteobacteria* (Gamma-class)	98.0/98.0	KR054069
**20**	*HB31*	Uncultured *Halovibrio* sp. clone BPS_CK84 [HQ857607] petroleum hydrocarbon contaminated gradient in a saline alkaline soil. /*Halovibrio* sp. K12-34B [KC309459] Isolated from temporary saline tidal pools.	*Proteobacteria* (Gamma-class)	98.0/98.0	KR054070
**20**	*HB65*	Halophilic bacterium MBIC3303 [AB015022] isolated from an halophilic lake. */Bacillus qingdaonensis* strain JCM 14087 [NR_113192] a moderately halophilic and alkalitolerant bacterium isolated from soil.	*Firmicutes*	98.0/97.0	KR054071
**20**	*HB67*	Uncultured *Halovibrio* sp. clone BPS_CK84 [HQ857607] petroleum hydrocarbon contaminated gradient in a saline alkaline soil. /*Halovibrio* sp. K12-34B [KC309459] Isolated from temporary saline tidal pools.	*Proteobacteria* (Gamma-class)	98.0/98.0	KR054072
**20**	*HB68*	*Marinococcus halophilus* strain AX18 [KJ873909] from soil samples of Qaidam Basin, China.	*Firmicutes*	99.0	KR054073
**20**	*HB69*	Halophilic bacterium MBIC3303 [AB015022] isolated from an halophilic lake. */Bacillus qingdaonensis* strain JCM 14087 [NR_113192] a moderately halophilic and alkalitolerant bacterium isolated from soil.	*Firmicutes*	98.0/97.0	KR054074
**20**	*HB70*	*Salicola* sp. ST_23S38, isolate ST_23S38 [LN650037] from sediments of solar salterns.	*Proteobacteria* (Gamma-class)	99.0	KR054075
**20**	*HA51*	*Halococcus salifodinae* strains [NR_113436, NR_119309] from separated permo-triassic salt deposits.	Archaea; *Euryarchaeota*;	99.0	KR054076
**20**	*HA71*	*Halococcus salifodinae* strains [NR_113436, NR_119309] from separated permo-triassic salt deposits.	*Euryarchaeota*;	98.0	KR054077
**20**	*HA72*, HA74, HA75	*Halorubrum* sp. [KP030743, LN649867] isolated from solar salterns	*Euryarchaeota*;	100	KR054078
**20**	*HA76*, HA77	*Halorubrum* sp. [KJ573439] isolated from solar salterns.	*Euryarchaeota*;	100	KR054082
**20**	*HA78*	*Halococcus salifodinae* strains [NR_113436, NR_119309] from separated permo-triassic salt deposits.	*Euryarchaeota*;	99.0	KR054084
**Sample 13216**					
**3**	*HB3*	*Psychrobacter* sp. KKDK-5 [KF741282] arsenic tolerant marine bacteria. */Psychrobacter namhaensis* strain SW-242 16S [NR_043141].	*Proteobacteria* (Gamma-class)	99.0/99.0	KR054085
**3**	*HB15*, *HB*2, HB22, HB35	*Bacillus safensis* strain Y39 [KF641818].	*Firmicutes*	99.0	KR054086
**10**	*HB1*, HB8	*Bacillus baekryungensis* strain QD41 [KF933695] halophilic and halotolerant bacteria isolated from marine sediments for producing extracellular hydrolytic enzymes.	*Firmicutes*	99.0	KR054087
**10**	*HB7*, HB37, HB20	*Marinococcus halophilus* strain AX17 [KJ873908] from soil samples of Qaidam Basin, China.	*Firmicutes*	99.0	KR054088
**10**	HB17, *HB50*	*Virgibacillus byunsanensis* strain ISL-24 [NR_116615] isolated from a marine solar saltern.	*Firmicutes*	98.0	KR054089
**10**	*HB36*	*Marinococcus halophilus* strain AX17 [KJ873908] from soil samples of Qaidam Basin, China.	*Firmicutes*	99.0	KR054090
**10**	*HB39*	Uncultured bacterium clone SN180 [JQ825066] from Hexi corridor saline-alkali soil. /*Marinococcus luteus* strain KCTC 13214 [NR_114354] isolated from a mine of salt located in Tarija, Bolivia.	*Firmicutes*	99.0/99.0	KR054091
**20**	*HB24*	*Marinococcus halophilus* strain AX17 [KJ873908] from soil samples of Qaidam Basin, China.	*Firmicutes*	99.0	KR054092
**20**	*HB25*	Uncultured Gammaproteobacteria clone 2B4 [AY987840] in Maras salterns, a hypersaline environment in the Peruvian Andes. */Salicola* sp. ST_23S38, isolate ST_23S38 [LN650037] from sediments of solar salterns.	*Proteobacteria* (Gamma-class)	99.0/98.0	KR054093
**20**	*HB26*	Uncultured Gammaproteobacteria clone 2B4 [AY987840] in Maras salterns, a hypersaline environment in the Peruvian Andes. */Salicola* sp. ST_23S38, isolate ST_23S38 [LN650037] from sediments of solar salterns.	*Proteobacteria* (Gamma-class)	99.0/98.0	KR054094
**20**	*HB27*, HB53, HB58	Uncultured *Halovibrio* sp. clone BPS_CK84 [HQ857607] petroleum hydrocarbon contaminated gradient in a saline alkaline soil. /*Halovibrio* sp. K12-34B [KC309459] Isolated from temporary saline tidal pools.	*Proteobacteria* (Gamma-class)	98.0/98.0	KR054095
**20**	*HB54*	Uncultured bacterium clone SN180 [JQ825066] from Hexi corridor saline-alkali soil. /*Marinococcus halophilus* strain AX18 [KJ873909] from soil samples of Qaidam Basin, China.	*Firmicutes*	99.0/99.0	KR054096
**20**	*HB55*, HB60, HB63	Uncultured bacterium clone SN4 [EU735647] heavily oil-contaminated and pristine soils. */Halovibrio denitrificans* strain C4-2 [EU868859] halophilic and halotolerant bacteria isolated from a hypersaline pond in Sichuan, China.	*Proteobacteria* (Gamma-class)	98.0/98.0	KR054097
**20**	*HB62b*	*Salicola* sp. ST_23S38, isolate ST_23S38 [LN650037] from sediments of solar salterns.	*Proteobacteria* (Gamma-class)	99.0	KR054098
**20**	*HB64*	Uncultured *Halovibrio* sp. clone BPS_CK84 [HQ857607] petroleum hydrocarbon contaminated gradient in a saline alkaline soil. /*Halovibrio* sp. K12-34B [KC309459] Isolated from temporary saline tidal pools.	*Proteobacteria* (Gamma-class)	98.0/98.0	KR054099
**20**	*HA56*, *HA57* HA62	*Halorubrum* sp. [KP030743, LN649867] isolated from solar salterns.	Archaea; *Euryarchaeota*	100	KR054100KR054101
**20**	*HA61*	*Halorubrum* sp. [KP030743, LN649867] isolated from solar salterns.	*Euryarchaeota*	100	KR054102
**Contact plates (MEA)**					
**MEA**	*KPA*	*Kocuria salsicia* strain 104 [NR_117299] isolated from salt-fermented seafood.	*Actinobacteria*	99.0	KR054104
**MEA**	KPBa, *KPBb*	*Bacillus subtilis* strain BJ-17 [GQ280027] alkaliphilic bacteria isolated from effluent and sludge samples of soda ash industry.	*Firmicutes*	99.0	KR054105
**MEA**	*KPC*	Uncultured bacterium clones [GQ157084, GQ157081, GQ157077] bacteriology of pouchitis. */Pseudomonas rhizosphaerae* strain DSM 16299 [CP009533] a phosphate-solubilizing rhizobacterium for bacterial biofertilizer.	*Proteobacteria* (Gamma-class)	99.0/99.0	KR054106
**MEA**	*KPD*	*Bacillus simplex* strain 265XG8 [KF818647] an endophytic bacterium.	*Firmicutes*	99.0	KR054107

* Strains marked in italics were subjected to sequencing and phylogenetic analyses.

### Direct extraction and amplification of total DNA from the wooden material

Total DNA from the wooden material was extracted directly from small wood scrapes by using the FastDNA Spin Kit for Soil from MP Biomedicals (Illkrich, France) with slight modifications as described by Piñar *et al*. [23]. After the DNA extraction, the concentration and quality of the DNA extracts were assessed using a NanoDrop^®^ ND-1000 Spectrophotometer (peqLab Biotechnologie GmbH, Linz, Austria), according to the manufacturer’s protocol and in triplicate. The extracted DNA was used directly for PCR amplification. PCR analyses were executed using the same equipment and the same mastermix mentioned above.

For the amplification of fungal ITS regions, fragments of 450–600 bp, corresponding to the ITS1 and the ITS2 regions, and the 5.8S rRNA gene situated between them, were amplified with the primer pairs ITS1 forward and ITS4 reverse [18]. The thermocycling program was as follows: 5 min at 95°C, followed by 35 cycles of [95°C, 1 min; 55°C, 1 min and 72°C, 1 min] with a final extension step for 5 min at 72°C. For genetic fingerprints, the obtained PCR products were further amplified in a nested PCR using the ITS1 primer with an attached GC clamp on the 5’end and the reverse primer ITS2. The cycling scheme was the same as that described by Michaelsen *et al*. [24].

For the amplification of bacterial 16S rRNA fragments, the primer pair 341f/985r [25, 26] was used under the following thermocycling conditions: 5 min at 95°C, followed by 30 cycles consisting of [95°C, 1 min; 55°C, 1 min and 72°C, 1 min] followed by a final extension step for 5 min at 72°C. For DGGE-fingerprints, a semi-nested PCR was performed using the primers 341f-GC [25] and 518r [27]. PCR conditions were as described by Schabereiter-Gurtner *et al*. [28]. For the amplification of archaeal 16S rRNA fragments, the primers and thermocycling conditions were used as described above. For DGGE-fingerprints, a semi-nested PCR was performed using the archaeal specific primer ARC344 (forward) and the universal sequence 518 (reverse). The reverse primer contained at the 5’ end a 40-base GC-clamp [25]. PCR conditions were as described by Piñar *et al*. [29].

### DGGE analyses

DGGE was performed as previously described [25] using a D-Code system (Bio-Rad) in × 0.5 TAE [20 mM Tris, 10 mM acetate, 0.5 mM Na2 EDTA; pH 7.8 with 8% (w/v) acrylamide]. Gels were run at a constant temperature of 60°C with a voltage of 200 V for a period of 3.5 h for Bacteria and Archaea, and 5 h for fungal fingerprints. The linear chemical gradient of denaturants used in this study [100% denaturing solution contains 7 M urea and 40% (v/v) formamide] was 30–60% for the screening of Bacteria and Archaea, and 20–50% for the screening of fungal communities. After completion of electrophoresis, gels were stained in a 1 μg ml^-1^ ethidium bromide solution [stock: 10 mg ml^-1^] for 20 min and afterwards visualized by a UVP documentation system (Bio-Rad Transilluminator, Universal Hood; Mitsubishi P93D-printer).

### Creation of clone libraries, DGGE-screening and Sequencing analyses

For the construction of clone libraries, 2×3 μl DNA templates of each sample were amplified in 2×50 μl reaction volumes using the universal primers 341f/985r, ARC344/ARC915 and ITS1/ITS4 for the bacterial, archaeal and fungal DNA amplification, respectively, as mentioned above. The PCR products were purified using the QIAquick PCR Purification Kit Protocol (Qiagen, Hilden, Germany) and re-suspended in ddH_2_O water. Subsequently, a volume of 5.5 μl of the purified PCR product was ligated into the pGEM-T easy Vector system (Promega, Mannheim, Germany) following the manufacturer's instructions. The ligation products were transformed into One Shot TOP10 cells (Invitrogen). The identification of recombinants was performed as previously described by Sambrook *et al*. [30].

Fifty clones from each clone library were harvested and screened in a DGGE gel as described by Schabereiter-Gurtner *et al*. [28]. The band positions of the bacterial, archaeal and fungal clones were compared with each other and with the DGGE profile of the original samples. Inserts of clones matching the most intense bands and with faint bands of the DGGE fingerprint of the original were selected for sequencing. For sequencing of the selected clones, 100 μl PCR product generated with primers SP6 and T7 was purified with a QIAquick PCR Purification Kit (Qiagen) and externally sequenced by Sanger sequencing (GATC Biotech, Germany). Comparative sequence analyses was done by comparing pair-wise insert sequences with those available in the online database provided by the NCBI (National Center for Biotechnology Information), using the BLAST search program [22]. The ribosomal sequences of the bacterial, archaeal and fungal clones have been deposited at the NCBI nucleotide database under accession numbers from KR017784-KR017789 for Bacteria, from KR017790-KR017799 for Archaea, and from KR017800-KR017806 for Fungi.

## Results

### Cultivation analyses

#### Fungi

A total of 39 fungal strains (designated HF1-HF39) could be isolated from the three stair samples. Some isolates were found to be identical in morphology and appearance; therefore, only 26 isolates were finally selected for further analyses (Table 1). Originating from the sequenced isolates, 5 isolates (19%) were obtained from sample 425, 16 isolates (62%) from sample 1003 and 5 isolates (19%) from sample 13216. Concerning the isolation procedure, 19 strains were isolated from sampling with contact plates, which represented the major part of fungi exhibiting different morphological appearance; whereas seven additional strains were isolated by the direct plating of wood scrapes suspensions, as mentioned in the Methods section (Table 1). All strains were further cultivated in MEA or SA media. Results showed that most of the strains were capable of growing on MEA, with the exception of seven isolates (see Table 1), which could only grow on SA, containing 10% (w/v) NaCl.

To screen the fungal isolates for further characterization, the polymorphism of the cellobiohydrolase gene (*cbh*-I) [10] was used as genotyping strategy (S2 Fig). All strains were typeable by using this technique and most of them showed unique *cbh*-I profiles. Nevertheless, the strains HF29, HF31 and HF39, and separately strains HF33 and HF37 showed the same *cbh*-I profiles and could be genotypically clustered into two different genotypic groups, respectively. All strains were subjected to sequencing for phylogenetic identification as described in the Methods section. Table 1 shows the phylogenetic identification of the isolated fungal strains. The similarity values with sequences from the NCBI database ranged from 94 to 99%. Most of the isolates were affiliated with cultured fungal strains, but the strains HF1, HF33 and HF37 were mostly related (99% similarity) to an uncultured clone detected at an archaeological excavation site in Yicheng, Shanxi, China, and with less similarity (96%) with cultivated species of the genus *Phialosimplex*. The same was observed for the strains HF5 and HF16, which were most affiliated (99% similarity) to uncultured fungal clones (Table 1), and with less similarity (98%) with *Cladosporium* sp. strains and with *Verrucocladosporium dirinae*, respectively.

The results showed in Table 1 revealed that all 26 strains sequenced were affiliated with species of 14 different genera within the phyla *Ascomycota* (23 strains) and *Basidiomycota* (3 strains). Within the *Ascomycota* phylum, a total of eleven different genera were identified, being species of the genus *Phialosimplex* and *Aspergillus* the most abundant isolated strains from all samples in SA and MEA media, respectively. In addition, species of the genera *Penicillium*, *Cladosporium*, *Verrucocladosporium*, *Stachybotris*, *Engyodontium*, *Nectria*, *Brachycladium*, *Pseudotaeniolina* and *Exophiala* were growing on MEA medium. Within the *Basidiomycota* phylum, species belonging to three different genera were retrieved, two of them in MEA medium, *Sterigmatomyces* and *Phlebia*, and one in SA medium, namely *Wallemia*.

#### Bacteria and Archaea

From samples 1003 and 13216, three aliquots of the collected samples were used to perform conventional enrichment cultures on three different media (see the Methods section) at salinities ranging from 3 to 20% (w/v) NaCl. A total of 66 pure strains differing in morphology and appearance could be isolated from the enrichment cultures. Thereof, 37 were isolated from sample 1003 and 29 from sample 13216. Additionally, 5 strains were isolated from contact plates containing MEA medium (Table 2). Summarized over all 71 cultured strains, 13 isolates (18.3%) were isolated from media containing 3% (w/v) NaCl, 18 strains (25.4%) grew on 10% (w/v) NaCl media, 35 strains (49.3%) on 20% (w/v) NaCl media and 5 strains (7%) on MEA.

In order to make a pre-selection of the isolated strains to be sequenced and further characterized, all isolated strains were subjected to RAPD-PCR analyses (S3 Fig). The obtained RAPD profiles allowed the clustering of the strains into different groups. RAPD analyses enabled the selection of 7different fingerprints among the 13 strains isolated from the 3% (w/v) NaCl medium (S3a Fig and Table 2). The same was observed for the strains isolated from 10% (w/v) NaCl, allowing the clustering of 18 strains in 10 different RAPD profiles (S3b Fig and Table 2). When the strains isolated from sample 13216 in 20% (w/v) NaCl media were analyzed, a total of 10 different RAPD profiles were produced, being 8 RAPD groups comprising Bacteria and two of them comprising Archaea (S3c Fig and Table 2). For sample 1003, 19 strains isolated in 20% (w/v) NaCl media were clustered in 14 RAPD groups, being 10 RAPD groups identified as bacterial and 4 as archaeal members (S3d Fig and Table 2). Those strains isolated at different NaCl concentrations and showing different RAPD profiles were subjected to sequencing and identification (Table 2). Similarity values ranged from 97–99% for Bacteria and from 98–100% for Archaea.

Results derived from sequence analyses showed that 59 strains affiliated with bacterial species of 12 genera, belonging to three phyla: the *Proteobacteria*, of the Gamma-class, being identified as *Halovibrio* sp., *Idiomarina* sp., *Psychrobacter* sp., *Salicola* sp. and *Pseudomonas* sp.; the *Firmicutes*, being identified as *Staphylococcus* sp., *Bacillus* sp., *Virgibacillus* sp., *Marinococcus* sp. and *Planococcus* sp. and the *Actinobacteria*, with the species *Janibacter cremeus* and *Kocuria salsicia*. In addition, 12 strains affiliated with archaeal species of the phylum *Euryarchaeota*, namely with species of the genera *Halococcus* and *Halorubrum*.

### Molecular analyses

#### Total DNA Extraction from wood scrapes, Amplification, and DGGE Fingerprinting

DNA was extracted from the aliquots of each stair destined for molecular analyses, yielding a concentration of 4.2–18.45 ng DNA μl^−1^, and was further amplified by PCR with primers targeting the 16S rRNA of Bacteria and Archaea as well as the ITS regions of Fungi. The DGGE profiles obtained from the three different stairs are shown in S4a and S4b Fig (Bacteria and Archaea) and in S4c Fig (Fungi). The DGGE fingerprints derived from the three independent stairs were shown to be almost identical for Bacteria, Archaea and Fungi, respectively, showing a very homogeneous microbiota. Therefore, the extracted DNA of the three stairs samples was pooled for further cloning and phylogenetic analyses, as mentioned in the Methods section.

#### Phylogenetic Identification of the Microbial Communities colonizing the wooden staircase

To accomplish phylogenetic identification of the bacterial, archaeal and fungal communities inhabiting the wooden staircase, clone libraries containing the 16S rRNA of Bacteria and Archaea (one clone library each) or the ITS fragments (one clone library) were generated as mentioned in the Methods section. The obtained sequences were compared with those of known microorganisms in the NCBI database. The comparative sequence analyses revealed similarity values ranging from 98–99%, 93–99% and 97–100% with sequences from the NCBI database for Bacteria, Archaea and Fungi respectively.

Sequence analyses revealed that 100% of the bacterial 16S rRNA cloned sequences were members of the phylum *Proteobacteria*. All of them were shown to be most related (99% similarity) to an uncultured bacterium clone detected in the Ebinur Lake wetland and with less similarity (98%) with *Halovibrio* spp. isolated from temporary saline tidal pools. These results supported the dominance of *Halovibrio* sp. in Petri plates derived from the 10% and 20% enrichment cultures.

The archaeal 16S rRNA cloned sequences were all identified as members of the phylum *Euryarchaeota*, of the Halobacteria-class. They affiliated with species of five different genera, namely *Halolamina* (48.9% of the screened clones), *Halobacterium* (36.2%), *Halorubrum* (6.4%), *Halococcus* and *Haloplanus* (4.2% each). However, by cultivation assays only strains belonging to the genera *Halococcus* and *Halorubrum* could be isolated in media containing 20% (w/v) of NaCl.

Concerning fungi, 39.6% of the screened clones proved to be most related (99% similarity) to an uncultured clone detected at an archaeological excavation site in Yicheng, Shanxi, China, and with less similarity (97–98%) with cultivated species of the genus *Phialosimplex*. The rest of the screened clones (60.4%) were all most related (98–100% similarity) to *Phialosimplex salinarum*. These results supported those obtained by cultivation assays, being the dominant fungal isolates most related to members of the genus *Phialosimplex*.

## Discussion

Often, objects buried in the past under natural conditions have remained very well preserved until their discovery. However, the impact of excavation work, transport, storage, investigation and subsequent exhibition can lead to their deterioration. Some studies warn about the risk of excavating and exhibiting art objects [31, 32]. Furthermore, in cases where it cannot be ensured that a freshly excavated object can be preserved and protected against biodeterioration, it would be preferable for it to remain buried until better preservation methods are available [32].

This study concerns a 3100-year-old wooden staircase, which had been discovered in a salt mine in Austria. Researchers tried to maintain the staircase in the salt mine where it was discovered, but, because of the ongoing threat of earth movements in this area, they had to remove the staircase. Very special care was taken to relocate the staircase, step-by-step, to the NHM (Vienna), where each stair was stored independently. During the relocation of the stairs, fungal mycelia could be observed in the small gap between the wood and the sediment on which the staircase was laying. In addition, best practices were employed for the different scientific investigations performed in the museum on this unique object. Nevertheless, as soon as the stairs had been removed from their naturally preserving environment (high concentrations of NaCl in a salt mine), fungal mycelia started to develop on the wood and on the dark deposits on the surface. The knowledge of the nature of the colonizing microbiota was of great concern because future conservation treatments should be derived from the scientific investigations. To this end, we analyzed the micro-flora thriving on the wood and on the old patina in order to check for possible biodegradative microorganisms that might become active under the new storage conditions.

Microbiological analyses revealed the presence of microorganisms that were able to grow on the different supplied media. Even if we cannot rule out that some of the isolated microorganisms could be in a dormant stage at the time of sampling, the results obtained in this study clearly show that these microorganisms can actively grow when the requirements for growth are suitable. This point reveals the potential risk for the conservation of the staircase. If the naturally preserving environment that was surrounding the staircase for more than 3000 years changes, as it was the case when the staircase was temporarily located in the NHM, these microorganisms may become active and represent a real threat for the wooden staircase.

Concerning fungi, out of the 26 fungal strains that were characterized, nineteen grew on MEA, whereas 7 strains were shown to grow exclusively on media containing 10% (w/v) NaCl. The strain HF7 was identified as *Wallemia muriae*, which had previously been detected in house dust and indoor environments in Europe [33]. *Wallemia* is a ubiquitous genus usually isolated from xeric environments, including sweet and salty foods, soil and hypersaline water of salterns [34, 35]. Nevertheless, the species *W*. *sebi* and *W*. *muriae* are the two species of *Wallemia* most commonly isolated from the indoor environment, an arid niche where xerophiles are common [33, 35]. The other strains, growing only in 10% (w/v) NaCl media, were all members of the genus *Phialosimplex*. However, the *cbh*-I profiles (S2 Fig) as well as the sequence analyses (Table 1) indicated two different groups of these *Phialosimplex*-related strains. The first was comprised of the isolates HF1, HF33 and HF37, which were most related to an uncultured clone detected at an archaeological excavation in China (data not published). These strains may potentially represent a new species of the genus *Phialosimplex*. The second cluster was comprised of the strains HF29, HF31 and HF39, all of them most related to *Phialosimplex salinarum*. The polymorphisms produced by the amplification of the *cbh*-I gene demonstrated, once more, that this genotyping method is useful for the classification of fungal isolates and for the detection of the potential cellulolytic capabilities of fungi. The cellobiohydrolase gene was described for the first time by Kraková *et al*. [10] as a gene target to detect the potential cellulolytic ability of filamentous fungi.

The genus *Phialosimplex* was described for the first time in 2010, harbouring a species representing a pathogen for dogs [36]. Nevertheless, the recent discoveries of sequences most related to members of the genus *Phialosimplex* in saline environments by different authors [6, 23, 37, 38] shows that the diversity and the adaptation versatility displayed by members of this genus are wider than initially suspected and are worthy of further investigation. Concerning the species *P*. *salinarum*, strains of this species were recently isolated from a salt mine and were shown, indeed, to degrade cellulose and proteins, being established as a new species of the genus *Phialosimplex* [6]. Nevertheless, in a recent study, the genus *Phialosimplex* has been proposed as synonym of the genus *Aspergillus* [39]. The isolation of *Wallemia* and *Phialosimplex* species in this study is in accordance with the halophilic environment provided by the salt mine in Hallstatt.

It is worthy of mention that besides the *Phialosimplex*–related strains, species most related to the *Stachybotrys* and *Cladosporium* genera are known to possess high cellulolytic activity, and must be regarded as posing a threat to the wood. In addition, sequences related to a *Penicillium chrysogenum* strain that produces cellulases and hemicellulases were detected (Table 1).

The isolated bacterial and archaeal strains were genotypically discriminated by RAPD-PCR. The DNA profiles obtained allow discrimination at the subspecies level, based on the DNA diversity in the entire bacterial genome, offering a broad spectrum of genetic variation [17, 40]. Sequencing results showed that most of the isolated strains were moderately halophilic bacteria growing optimally in media containing 3–10% (w/v) salt, as the species of *Idiomarina*, *Psychrobacter* [41], *Kocuria* [42] *Staphylococcus*, *Virgibacillus* [43] *Planococcus* [44] and *Janibacter* [45], and some species of the genera *Bacillus*. Interestingly, some strains were isolated only in media containing the highest NaCl concentrations (10–20% (w/v) NaCl), as those related to species of *Halovibrio*, *Salicola*, *Marinococcus* and some species of *Bacillus* [46]. Moderately halophilic bacteria grow optimally in media containing 3–15% (w/v) salt, but they can also grow in concentrations as high as 25% [47]. This is the case for the species isolated in this study. In addition, the halophilic archaeal species of the *Halococcus* and *Halorubrum* genera were isolated exclusively in media containing 20% (w/v) NaCl [48]. In summary, all bacterial and archaeal strains were shown to be autochthonous microorganisms typical of the environment provided by the salt mine.

The role of moderately halophilic bacteria in the degradation of wood has been given less attention, but there are several studies showing the cellulolytic activity of this group of bacteria, as *S*. *equorum* [49] and *Bacillus* strains [50], both detected in this study, as well as the production of extracellular hydrolytic enzymes by moderately halophilic microorganisms [50, 51]. Furthermore, the role that the detected Archaea could play in the degradation of the staircase should not be overlooked. So far, few archaeal cellulases have been reported [52, 53]. Nevertheless, Birbir *et al*. [54] detected cellulase activity in different archaeal strains isolated from the Tuzkoy salt mine in Turkey, belonging to the genera *Haloarcula*, *Natrinema*, *Halobacterium* and *Halorubrum*, being the last two genera also detected in this study. Similarly, the same authors found archaeal isolates showing cellulase activity in the Tuz Lake and the Kaldirim and Kayacik salterns in Turkey [55], and they postulated that cellulase activity might thus be a common property of haloarchaeal strains. However, whether such cellulase-positive archaea are indeed able to grow on cellulose as carbon and energy source remains unknown.

Summarizing, our results indicate that some of the bacteria and archaea detected in this study also qualify as potential candidates as deteriorative agents of the wood.

## Conclusions

The results obtained in this study highlight that microbiological and molecular analyses complemented each other for the evaluation of the microbial community dwelling on the 3100 year old staircase excavated at Hallstatt.

Microbiological analyses showed that the surface of the stairs—including the old biofilms- were contaminated with common airborne fungi, but the dominant members, identified as halophilic strains of the genus *Phialosimplex* (synonym: *Aspergillus*), were shown to be the part of the autochthonous mycobiota originating from the material and the salty environment. Unfortunately, besides *Phialosimplex* sp., some other species with potentially cellulolytic capabilities, such as *Stachybotrys* and *Cladosporium*, were present and must be regarded as a threat to the wood.

The bacteria and archaea identified in the wooden staircase were shown to be moderately halophilic and halophilic members of these domains adapted to the salty conditions present in the salt mine from which they originated. Some of them were phylogenetically related to some cellulose-degrader counterparts, being also potential candidates as deteriorative agents of the wood.

Finally, and due to the evidence that the detected microorganisms were able to grow actively under suitable environmental conditions, the conservator-restorers and wood experts from the University of Natural Resources and Life Sciences, Vienna, decided to rehouse the staircase in a dry environment. Several tests and analysis indicated that the salt contained in the prehistoric wood is crystallized and, therefore, stable beneath relative humidity of 75%. The single parts of the staircase have been carefully dried by a special adapted method by the Institute of Wood Technology and Renewable Materials, University of Natural Resources and Life Sciences, Vienna, and afterwards in February 2015, the staircase was reassembled in a huge showcase 100 meters below ground in a therefor newly built gallery. This gallery is part of the guided tours in the “Salzwelten”, Hallstatt. The staircase is separated from the visitors by an airtight transparent foil, and is stored by constant conditions: 8–9°C and 69–72% relative humidity. The climate in the showcase, and in the visitor area is permanently monitored by several measuring sensors. Until July 2015 no fungal mycelia could be observed on the staircase.

## Supporting Information

S1 Fig Map showing the area where the prehistoric staircase was discovered. Salt mine “Christian von Tusch Werk”, Austrian region of “Salzkammergut” (Upper Austria). The brown colour indicates the location where the staircase was buried.
(TIF)

S2 Fig PCR-profiles of the 26 characterized fungal strains derived from the amplification of the *cbh*-I gene. Lane M1: 100 bp ladder (Fermentas); lane 1: strain HF1; lane 2: strain HF2; lane 3: strain HF3; lane 4: strain HF4; lane 5: strain HF5; lane 6: strain HF6; lane 10: strain HF10; lane 11: strain HF11; lane 12: strain HF12; lane 13: strain HF13; lane 14: strain HF14; lane 16: strain HF16; lane 19: strain HF19; lane 20: strain HF20; lane 21: strain HF21; lane 22: strain HF22; lane 24: strain HF24; lane 25: strain HF25; lane 29: strain HF29; lane 31: strain HF31; lane 33: strain HF33; lane 37: strain HF37; lane 39: strain HF39; lane 8: strain HF8; lane 7: strain HF7; lane 9: strain HF9; M2: 1Kb ladder (Fermentas).
(TIF)

S3 Fig RAPD-PCR patterns of representative strains. Strains were isolated from 3% (**A**), 10% (**B**), 20% (w/v) NaCl, sample 13216 (**C**) and 20% (w/v) NaCl media, sample 1003 (**D**). The number of lanes indicates the number of the strains. M1: 100 bp ladder (Fermentas), M2: 1Kb ladder (Fermentas).
(TIF)

S4 Fig DGGE-fingerprints. Fingerprints derived from bacterial (**A**), archaeal (**B**) and fungal (**C**) communities colonizing all three stairs sampled. The linear chemical gradient of denaturants used was 30–60% for Bacteria and Archaea, and 20–50% for Fungi. Lane 1: stair 13216; lane 2: stair 1003; lane 3: stair 425; B: positive control Bacteria; A: positive control Archaea; F: positive control Fungi.
(TIF)
