# Supplementary figures and images for: Biodeterioration Risk Threatens the 3100 Year Old Staircase of Hallstatt (Austria): Possible Involvement of Halophilic Microorganisms
Source: PLoS One. 2016 Feb 17;11(2):e0148279. doi: 10.1371/journal.pone.0148279 (PMC4757552; doi:10.1371/journal.pone.0148279)

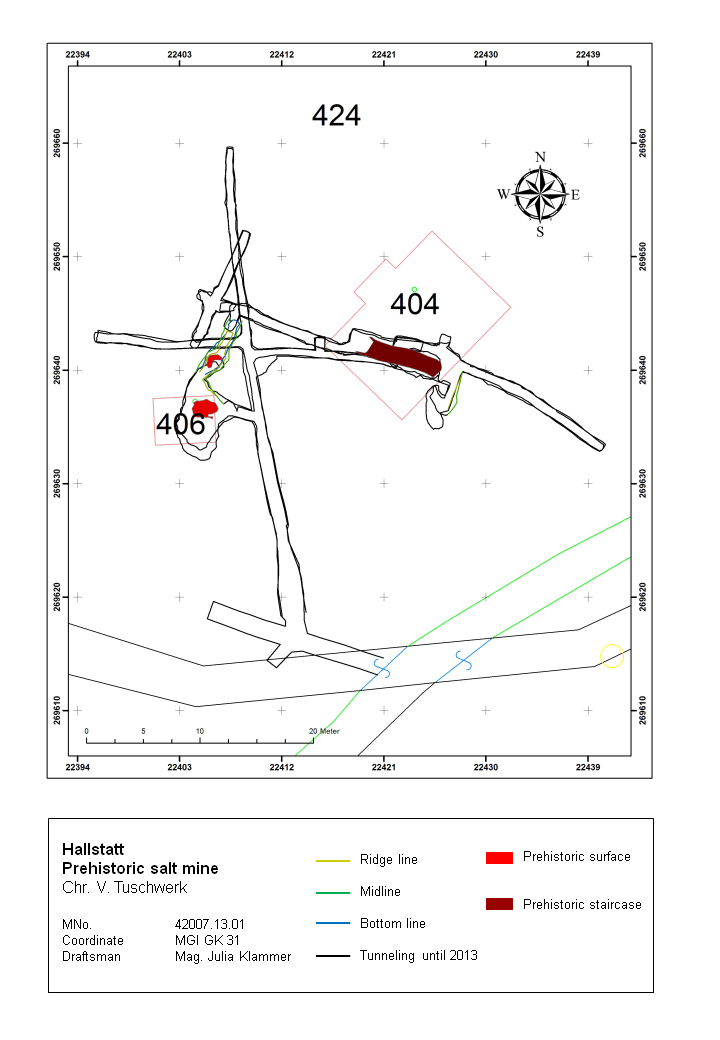

Supplement: S1 Fig — Salt mine “Christian von Tusch Werk”, Austrian region of “Salzkammergut” (Upper Austria). The brown colour indicates the location where the staircase was buried. (TIF) [file pone.0148279.s001.tif]

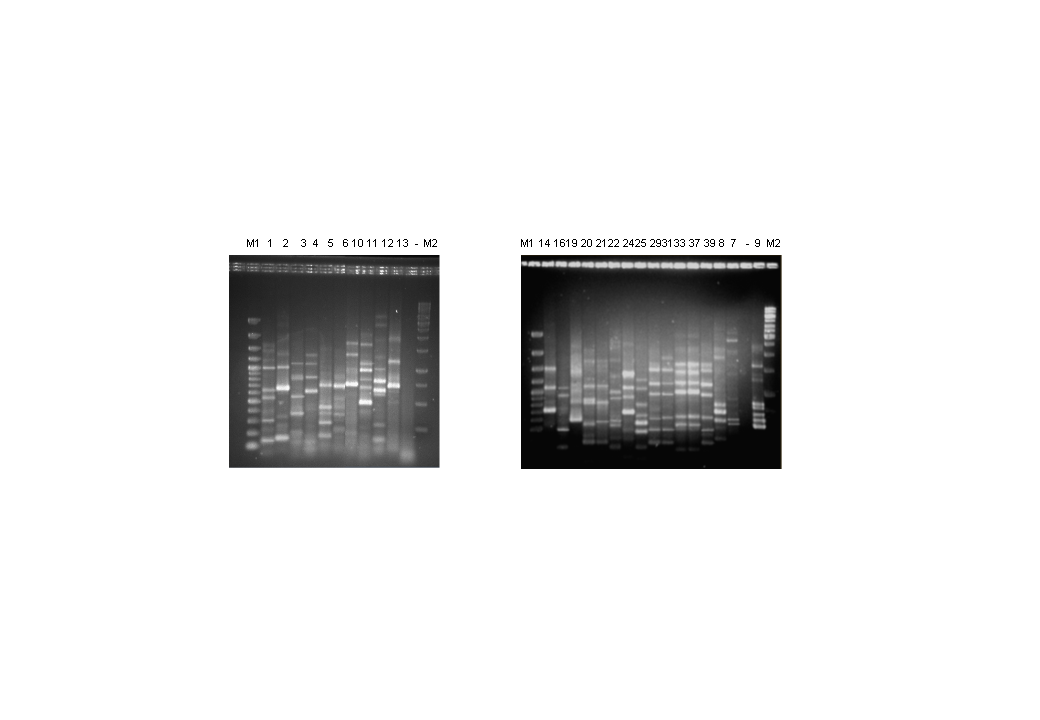

Supplement: S2 Fig — Lane M1: 100 bp ladder (Fermentas); lane 1: strain HF1; lane 2: strain HF2; lane 3: strain HF3; lane 4: strain HF4; lane 5: strain HF5; lane 6: strain HF6; lane 10: strain HF10; lane 11: strain HF11; lane 12: strain HF12; lane 13: strain HF13; lane 14: strain HF14; lane 16: strain HF16; lane 19: strain HF19; lane 20: strain HF20; lane 21: strain HF21; lane 22: strain HF22; lane 24: strain HF24; lane 25: strain HF25; lane 29: strain HF29; lane 31: strain HF31; lane 33: strain HF33; lane 37: strain HF37; lane 39: strain HF39; lane 8: strain HF8; lane 7: strain HF7; lane 9: strain HF9; M2: 1Kb ladder (Fermentas). (TIF) [file pone.0148279.s002.tif]

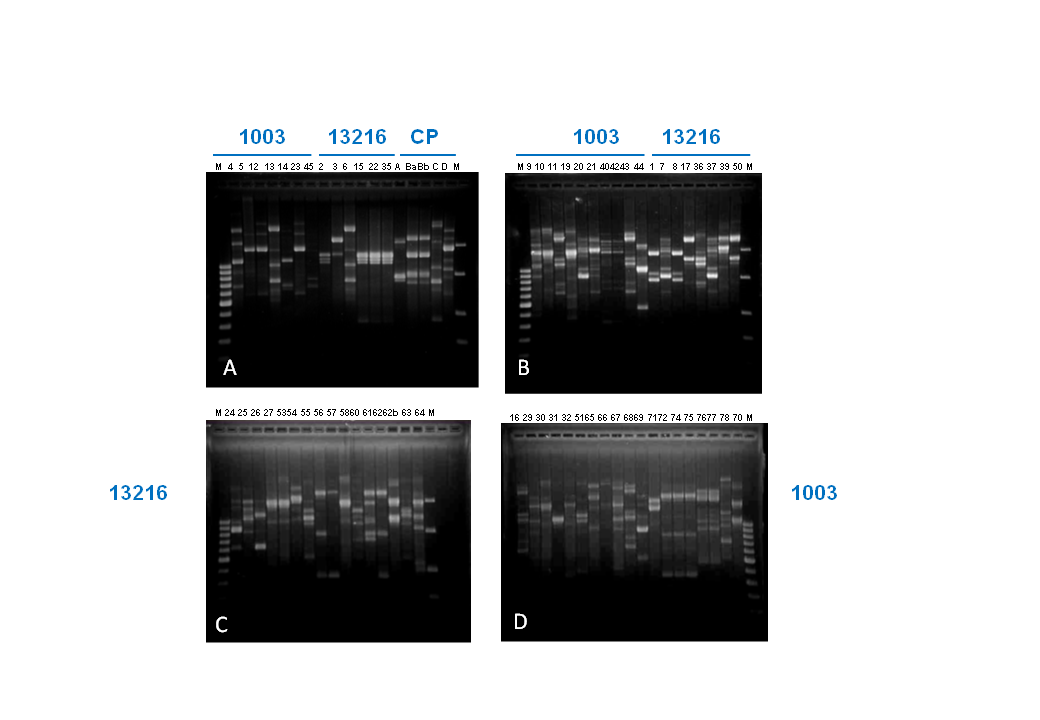

Supplement: S3 Fig — Strains were isolated from 3% (A), 10% (B), 20% (w/v) NaCl, sample 13216 (C) and 20% (w/v) NaCl media, sample 1003 (D). The number of lanes indicates the number of the strains. M1: 100 bp ladder (Fermentas), M2: 1Kb ladder (Fermentas). (TIF) [file pone.0148279.s003.tif]

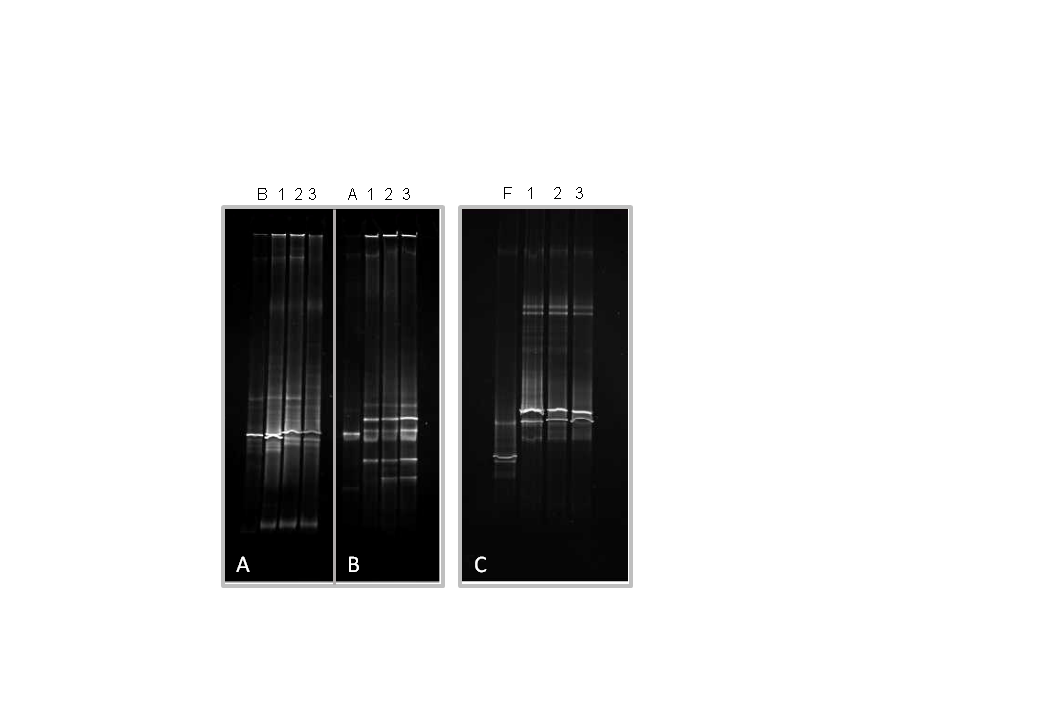

Supplement: S4 Fig — Fingerprints derived from bacterial (A), archaeal (B) and fungal (C) communities colonizing all three stairs sampled. The linear chemical gradient of denaturants used was 30–60% for Bacteria and Archaea, and 20–50% for Fungi. Lane 1: stair 13216; lane 2: stair 1003; lane 3: stair 425; B: positive control Bacteria; A: positive control Archaea; F: positive control Fungi. (TIF) [file pone.0148279.s004.tif]
